# Crystal structure and Hirshfeld surface analysis of 2,5-di­imino-8a-methyl-4,9-bis­(4-methyl­phen­yl)-7-oxo-6-phenyl-deca­hydro-2*H*-3,8-methano­pyrano[3,2-*c*]pyridine-3,4a-dicarbo­nitrile *N*,*N*-di­methyl­formamide monosolvate

**DOI:** 10.1107/S2056989023001718

**Published:** 2023-03-02

**Authors:** Farid N. Naghiyev, Victor N. Khrustalev, Anton P. Novikov, Mehmet Akkurt, Ali N. Khalilov, Ajaya Bhattarai, İbrahim G. Mamedov

**Affiliations:** a Department of Chemistry, Baku State University, Z. Khalilov str. 23, Az, 1148, Baku, Azerbaijan; b Peoples’ Friendship University of Russia (RUDN University), Miklukho-Maklay St.6, Moscow, 117198, Russian Federation; c N. D. Zelinsky Institute of Organic Chemistry RAS, Leninsky Prosp. 47, Moscow, 119991, Russian Federation; d Department of Physics, Faculty of Sciences, Erciyes University, 38039 Kayseri, Türkiye; e "Composite Materials" Scientific Research Center, Azerbaijan State Economic University (UNEC), H. Aliyev str. 135, Az 1063, Baku, Azerbaijan; f Department of Chemistry, M.M.A.M.C (Tribhuvan University) Biratnagar, Nepal; Vienna University of Technology, Austria

**Keywords:** crystal structure, hydrogen-bonding, C—H⋯π inter­actions, bi­cyclo [3.3.1]nonane ring system, Hirshfeld surface analysis

## Abstract

In the title compound, C_32_H_29_N_5_O_2_·C_3_H_7_NO, the bi­cyclo[3.3.1]nonane ring sys­tem adopts a half-chair/twist-boat conformation, with the phenyl rings in equatorial orientations with respect to the piperidine ring. The two oxane rings of the 2-oxabi­cyclo­[2.2.2]octane ring system exhibit a distorted boat conformation. Inter­molecular C—H⋯O and C—H⋯N hydrogen bonds connect the mol­ecules in the crystal, generating layers extending parallel to (100). These layers are connected by C—H⋯π inter­actions. A Hirshfeld surface analysis was per­formed to qu­antify the contributions of the different inter­molecular inter­actions, indicating that the most important contributions to the crystal packing are from H⋯H (52.5%), N⋯H/H⋯N (19.2%), C⋯H/H⋯C (18.8%) and O⋯H/H⋯O (8.3%) inter­actions.

## Abstract 2

Inter­molecular C—H⋯O and C—H⋯N hydrogen bonds connect individual mol­ecules into layers extending parallel to (100). These layers are connected by C—H⋯π inter­actions.

## 1. Chemical context

**Figure 1:**
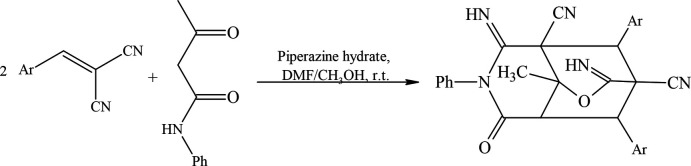
Chemical scheme for the one-pot synthesis of tricyclic pyrano[3,2-*c*]pyridine derivatives.

Different C—C, C—N, and C—O bond-formation methods play important roles in various organic synthesis directions (Aliyeva *et al.*, 2011 ▸; Zubkov *et al.*, 2018 ▸; Viswanathan *et al.*, 2019 ▸; Mamedov *et al.*, 2022 ▸). Heterocyclic systems, especially those comprising the pyrano[3,2-*c*]pyridine scaffold, are present in many natural or synthetic products with a wide spectrum of biological properties, such as anti­tumor, anti­tubercular, cholinesterase inhibitor and anti-diabetic activities (Mamedov *et al.*, 2019 ▸; Kumari *et al.*, 2018 ▸). One of the most effective synthetic approaches to these polyfunctional heterocyclic systems is a Michael addition of active methyl­ene compounds at the yl­idene malono­nitrile functionality (Girgis *et al.*, 2015 ▸). In a recent study (Mamedov *et al.*, 2019 ▸), we found that the reaction of two moles of aryl­idene malono­nitriles with acetoacetanilide in the presence of piperazine hydrate leads to the formation of novel tricyclic pyrano[3,2-*c*]pyridine derivatives at room temperature (Fig. 1 ▸).

In this context and with respect to our on-going structural studies (Naghiyev *et al.*, 2020 ▸, 2021 ▸, 2022 ▸; Khalilov *et al.*, 2022 ▸), we report here the crystal structure and Hirshfeld surface analysis of 2,5-di­imino-8a-methyl-4,9-bis­(4-methyl­phen­yl)-7-oxo-6-phenyl-deca­hydro-2*H*-3,8-methano­pyrano[3,2-*c*]pyridine-3,4a-dicarbo­nitrile *N*,*N*-di­methyl­formamide monosolv­ate, C_32_H_29_N_5_O_2_·C_3_H_7_NO. 
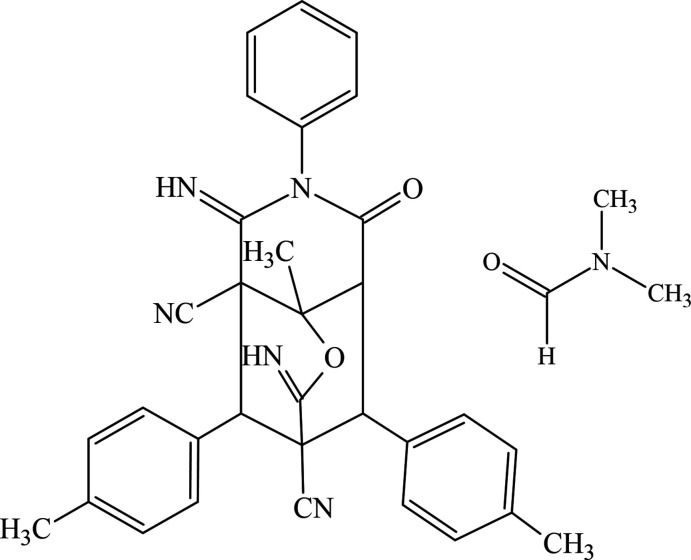


## 2. Structural commentary

**Figure 2:**
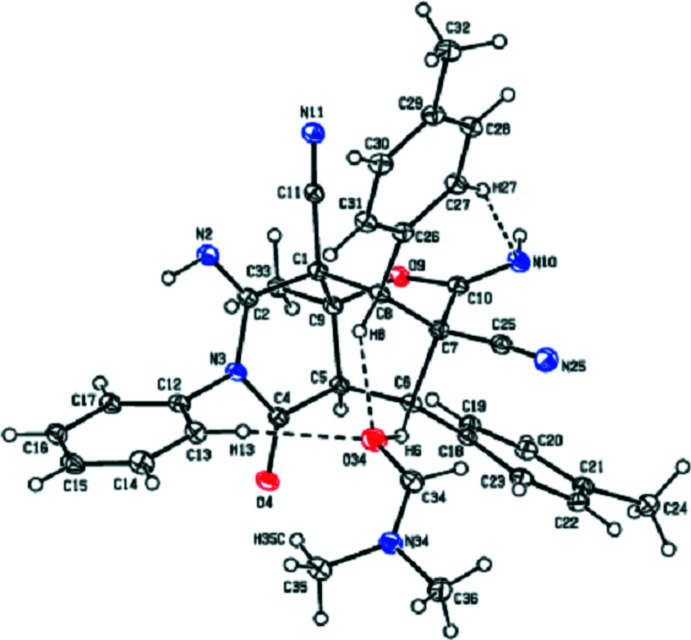
The mol­ecular entities of the title compound, showing the atom labelling and displacement ellipsoids drawn at the 30% probability level. C—H⋯O and C—H⋯N hydrogen bonds are indicated by dashed lines.

**Figure 3:**
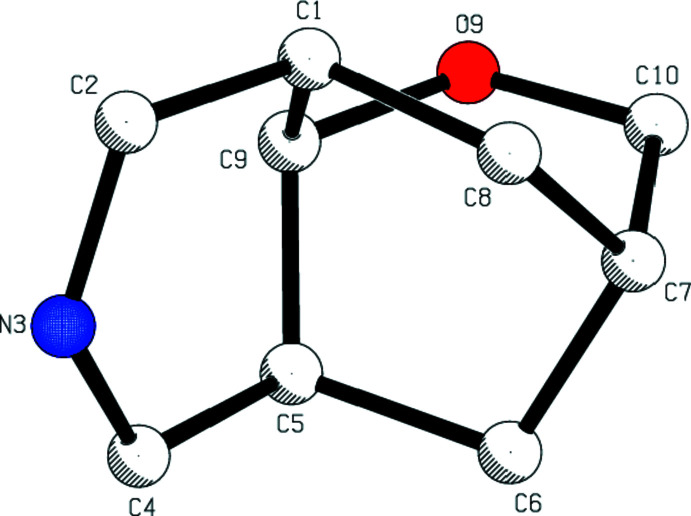
View of the octa­hydro-2*H*-3,8-methano­pyrano[3,2-*c*]pyridine ring sytem of the title compound.

The mol­ecular structure of the title compound is displayed in Fig. 2 ▸. The mol­ecular conformation is stabilized by an intra­molecular C—H⋯N hydrogen bond (Table 1 ▸) and consolidated by inter­molecular C—H⋯O inter­actions involving the *N*,*N*-di­methyl­formamide solvent mol­ecule (Fig. 2 ▸). As shown in Fig. 3 ▸, the bi­cyclo­[3.3.1]nonane ring system (C2/N3/C4–C8/C1/C9) adopts a half-chair/twist-boat conformation; the puckering parameters (Cremer & Pople, 1975 ▸) are *Q*_T_ = 0.529 (2) Å, θ = 53.0 (2)°, φ = 160.1 (3)° for the (N3/C2/C1/C9/C5/C4) ring, and *Q*_T_ = 0.889 (2) Å, θ = 89.21 (13)°, φ = 289.11 (14)° for the (C1/C8/C7/C6/C5/C9) ring. The phenyl rings (C12–C17, C18–C23 and C26–C31) are in equatorial orientations with respect to the piperidine ring (C1/C2/N3/C4/C5/C9). The two oxane rings (O9/C9/C1/C8/C7/C10 and O9/C9/C5/C6/C7/C10) of the 2-oxabi­cyclo­[2.2.2]octane ring system (C10/O9/C9/C1/C8/C7/C6/C5) exhibit a distorted boat conformation with puckering parameters *Q*_T_ = 0.799 (2) Å, θ = 91.88 (14)°, φ = 247.89 (15)° for the O9/C9/C1/C8C7/C10 ring, and *Q*_T_ = 0.826 (2) Å, θ = 96.04 (14)°, φ = 50.59 (15)° for the O9/C9/C5/C6/C7/C10 ring.

## 3. Supra­molecular features and Hirshfeld surface analysis

**Figure 4:**
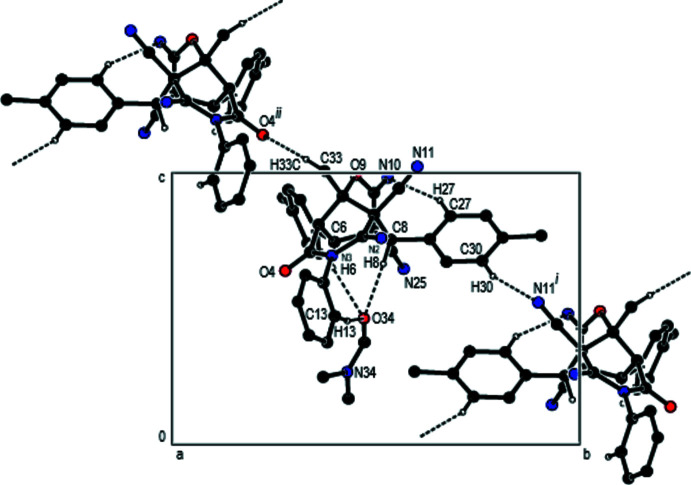
A partial view of the crystal packing along the *a* axis of the title compound with C—H⋯O and C—H⋯N hydrogen bonds indicated (dashed lines). [Symmetry codes: (i) *x*, −*y* + 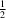 , *z* − 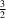 ; (ii) *x*, −*y* − 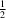 , *z* − 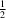 ].

**Figure 5:**
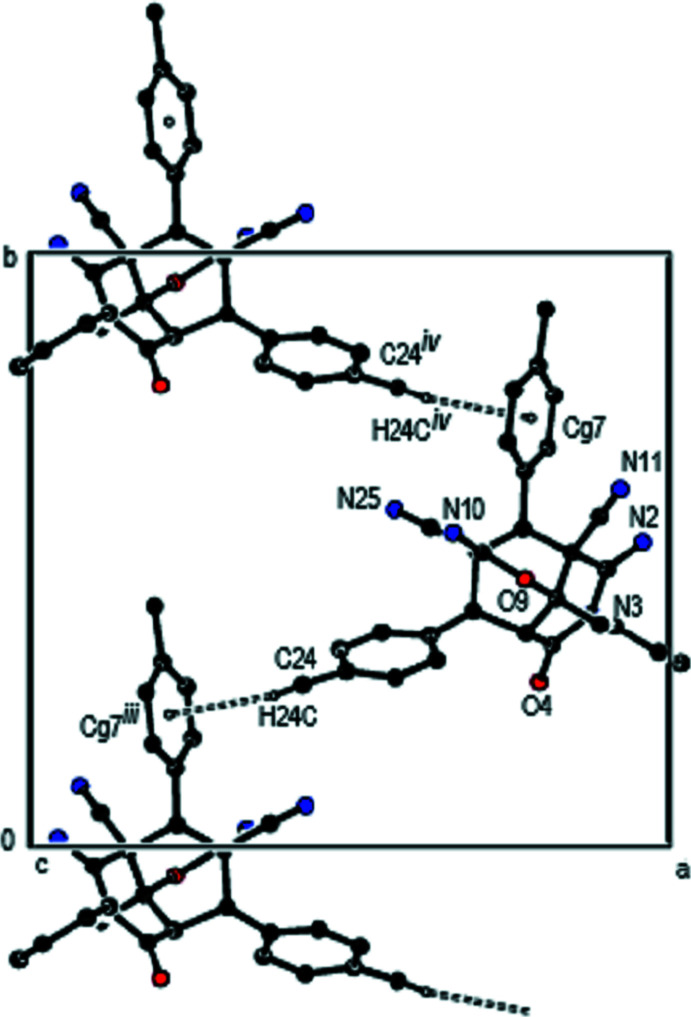
A general view of the packing in the unit cell of the title compound with C—H⋯π inter­actions indicated (dashed lines). [Symmetry codes: (iii) −*x* + 1, *y* − 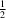 , −*z* + 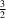 ; (iv) −*x* + 1, *y* + 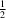 , −*z* + 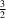 ].

In the crystal, inter­molecular C—H⋯O and C—H⋯N hydrogen bonds (Table 1 ▸) link individual mol­ecules, forming layers parallel to (100) (Fig. 4 ▸). These layers are connected by C—H⋯π inter­actions (Fig. 5 ▸). Inter­estingly, the imine C=N—H groups are not involved in hydrogen-bonding inter­actions.

**Figure 6:**
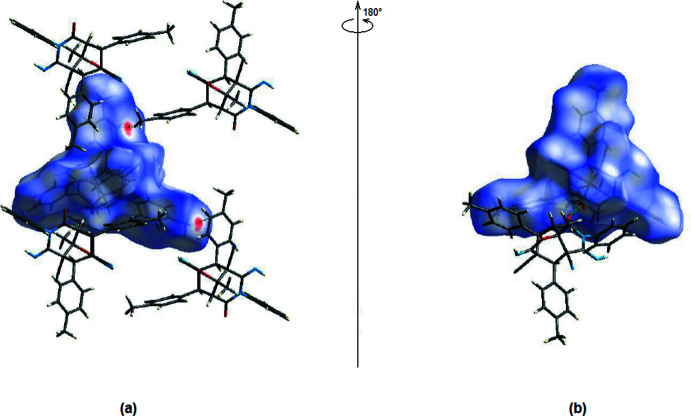
(*a*) Front and (*b*) back sides of the three-dimensional Hirshfeld surface of the title compound mapped over *d*_norm_, with a fixed colour scale of −0.1713 to 1.4361 a.u..

A Hirshfeld surface analysis was performed to qu­antify the inter­molecular inter­actions; the accompanying two-dimensional fingerprint plots were obtained using *CrystalExplorer17* (Turner *et al.*, 2017 ▸). The Hirshfeld surface mapped over *d*_norm_ using a standard surface resolution with a fixed colour scale of −0.1713 (red) to 1.4361 (blue) a.u. is shown in Fig. 6 ▸. The shorter and longer contacts are indicated as red and blue spots, respectively, on the Hirshfeld surfaces, and contacts with distances approximately equal to the sum of the van der Waals radii are represented as white spots. The most important red spots on the *d*_norm_ surface represent the aforementioned C—H⋯O and C—H⋯N inter­actions (Tables 1 ▸, 2 ▸).

**Figure 7:**
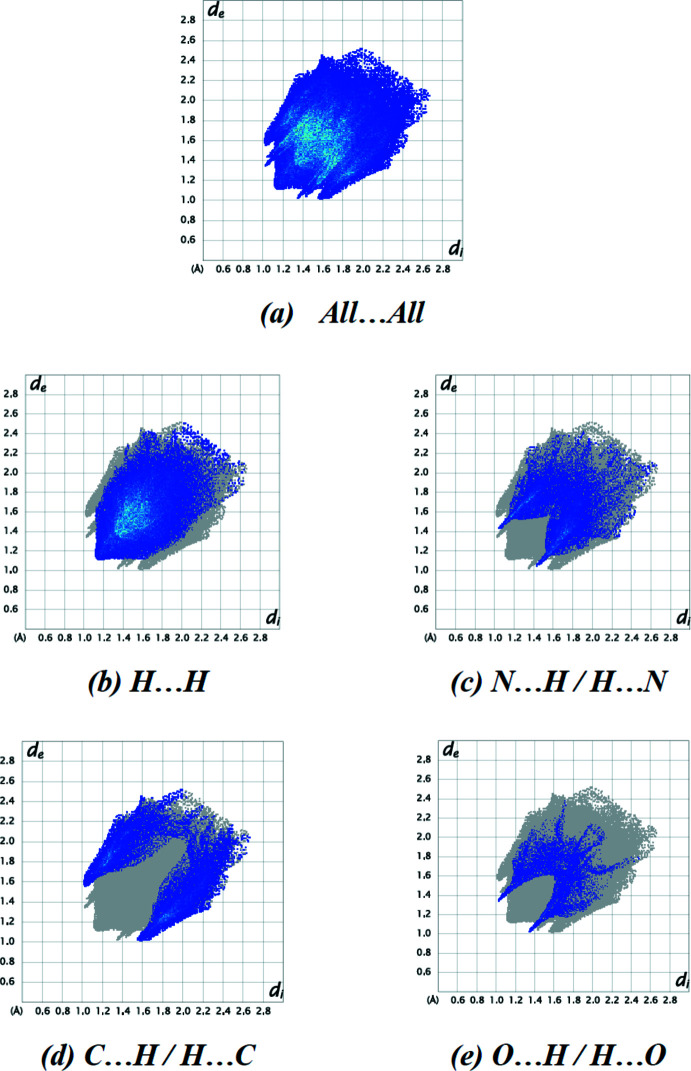
The two-dimensional fingerprint plots of the title compound, showing (*a*) all inter­actions, and delineated into (*b*) H⋯H, (*c*) N⋯H/H⋯N, (*d*) C⋯H/H⋯C and (*e*) O⋯H/H⋯O inter­actions. [*d*_e_ and *d*_i_ represent the distances from a point on the Hirshfeld surface to the nearest atoms outside (external) and inside (inter­nal) the surface, respectively].

Fig. 7 ▸ depicts the two-dimensional fingerprint plots of (*d*_i_, *d*_e_) points from all the contacts contributing to the Hirshfeld surface analysis in normal mode for all atoms. The most important inter­molecular inter­actions are H⋯H contacts, contributing 52.5% to the overall crystal packing. Other inter­actions and their respective contributions are N⋯H/H⋯N (19.2%), C⋯H/H⋯C (18.8%), O⋯H/H⋯O (8.3%), N⋯N (0.6%), C⋯N/N⋯C (0.3%), C⋯C (0.2%) and C⋯O/O⋯C (0.1%), respectively.

The Hirshfeld surface study verifies the significance of H-atom inter­actions in the packing formation. The contributions of H⋯H and N⋯H/H⋯N inter­actions imply that van der Waals inter­actions are important in the crystal packing (Hathwar *et al.*, 2015 ▸).

## 4. Database survey

The five most similar compounds found in a search of the Cambridge Structural Database (CSD, Version 5.42, update of September 2021; Groom *et al.*, 2016 ▸) for the bi­cyclo [3.3.1]nonane ring system are: 7-*tert*-butyl-*N*-methyl-2,4- diphenyl-3-aza­bicyclo­[3.3.1]nonane (**I**) (Kumaran *et al.*, 1999 ▸), *N*-acetyl-2,4-diphenyl-3-aza­bicyclo­[3.3.1]nonane (**II**) (Kumar­an *et al.*, 1999 ▸), *N*-methyl-2,4-bis­(2- methyl­phen­yl)-3-aza­bicyclo­[3.3.1]nonan-9-ol (**III**) (Kumaran *et al.*, 1999 ▸), 3-aza­bicyclo­[3.3.1]nonane- 2,4-dione (form 2) (**IV**) (Hulme *et al.*, 2006 ▸) and 2,4-bis­(furan-2-yl)-1,5-dimethyl-3-aza­bicyclo [3.3.1]nonan-9-one (**V**) (Venkateswaramoorthi *et al.*, 2013 ▸).

Compounds (**I**) and (**III**) crystallize in monoclinic space groups (*P*2_1_/*c*, *Z* = 4, and *P*2_1_/*n*, *Z* = 4, respectively), whereas (**II**) is ortho­rhom­bic (*Pbca*, *Z* = 8). In each of the three structures, the bicyclic ring system adopts a chair/chair conformation and the phenyl rings are in equatorial orientations with respect to the piperidine ring. In (**II**), apart from van der Waals forces, only weak inter­molecular C—H⋯O-type inter­actions are involved in the packing.

The structure of (**IV**) has monoclinic symmetry (*P*2_1_/*c*, *Z* = 8) and has two mol­ecules in the asymmetric unit. A 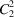 (8) chain motif (Bernstein *et al.*, 1995 ▸) is formed *via* N—H⋯O hydrogen bonds.

In (**V**), which likewise is monoclinic (*C*2/*c*, *Z* = 8), the bicyclic ring system adopts a twin-chair conformation. The two methyl groups attached to the bicycle are in an equatorial orientation for both rings. In the crystal, very long N—H⋯O hydrogen bonds connect the mol­ecules into a chain perpendicular to [010].

## 5. Synthesis and crystallization

The title compound was synthesized using a previously reported procedure (Mamedov *et al.*, 2019 ▸). Colourless crystals were obtained upon recrystallization from an ethanol/water (3:1 *v*/*v*) solution.

## 6. Refinement

Crystal data, data collection and structure refinement details are summarized in Table 3 ▸. All C-bound H atoms were placed at calculated positions and refined using a riding model, with C—H = 0.95–1.00 Å, and with *U*_iso_(H) = 1.2 or 1.5*U*_eq_(C). The N-bound H atoms were located from difference-Fourier maps and refined with free atomic coordinates and *U*_iso_ = 1.2*U*_eq_(N).

## Supplementary Material

Crystal structure: contains datablock(s) I. DOI: 10.1107/S2056989023001718/wm5672sup1.cif

Structure factors: contains datablock(s) I. DOI: 10.1107/S2056989023001718/wm5672Isup2.hkl

Supporting information file. DOI: 10.1107/S2056989023001718/wm5672Isup3.cml

CCDC reference: 2244417

Additional supporting information:  crystallographic information; 3D view; checkCIF report

## Figures and Tables

**Table 1:** Hydrogen-bond geometry (Å, °) *Cg*7 is the centroid of the C26–C31 benzene ring.

*D*—H⋯*A*	*D*—H	H⋯*A*	*D*⋯*A*	*D*—H⋯*A*
C6—H6⋯O34	1.00	2.44	3.297 (3)	143
C8—H8⋯O34	1.00	2.34	3.241 (3)	149
C13—H13⋯O34	0.95	2.47	3.373 (3)	159
C27—H27⋯N10	0.95	2.56	3.432 (3)	152
C30—H30⋯N11^i^	0.95	2.61	3.515 (3)	160
C33—H33*C*⋯O4^ii^	0.98	2.48	3.447 (3)	170
C24—H24*C*⋯*Cg*7^iii^	0.98	2.84	3.715 (2)	148

Symmetry codes: (i) 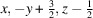 ; (ii) 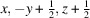 ; (iii) 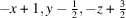 .

**Table 2:** Summary of short inter­atomic contacts (Å) in the title compound

Atoms	Distance	Symmetry code
O4⋯H33*C*	2.48	*x*, 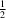 − *y*, − 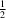 + *z*
H10*N*⋯H35*A*	2.35	*x*, *y*, 1 + *z*
H10*N*⋯H32*B*	2.52	*x*, 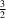 − *y*, 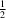 + *z*
H33*A*⋯N2	2.70	2 − *x*, 1 − *y*, 2 − *z*
H15⋯N2	2.92	2 − *x*, 1 − *y*, 1 − *z*
H24*A*⋯N10	2.82	1 − *x*, 1 − *y*, 2 − *z*
C29⋯H16	2.85	2 − *x*, 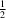 + *y*, 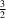 − *z*
C28⋯H24*C*	2.68	1 − *x*, 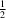 + *y*, 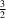 − *z*
N25⋯H36*C*	2.77	1 − *x*, 1 − *y*, 1 − *z*
H13⋯H35*C*	2.29	*x*, *y*, *z*
C18⋯H35*B*	2.91	*x*, 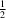 − *y*, 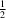 + *z*
H32*B*⋯C34	3.04	*x*, 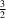 − *y*, 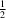 + *z*

**Table 3:** Experimental details

Crystal data	
Chemical formula	C_32_H_27_N_5_O_2_·C_3_H_7_NO
*M*_r_	586.68
Crystal system, space group	Monoclinic, *P*2_1_/*c*
Temperature (K)	100
*a*, *b*, *c* (Å)	17.6747 (3), 15.7656 (2), 10.9086 (2)
β (°)	105.666 (2)
*V* (Å^3^)	2926.79 (9)
*Z*	4
Radiation type	Cu *K*α
μ (mm^−1^)	0.70
Crystal size (mm)	0.14 × 0.11 × 0.08

Data collection	
Diffractometer	XtaLAB Synergy, Dualflex, HyPix
Absorption correction	Multi-scan (*CrysAlis PRO*; Rigaku OD, 2021 ▸)
*T*_min_, *T*_max_	0.900, 0.936
No. of measured, independent and observed [*I* > 2σ(*I*)] reflections	31226, 6140, 5275
*R*_int_	0.051
(sin θ/λ)_max_ (Å^−1^)	0.638

Refinement	
*R*[*F* ^2^ > 2σ(*F* ^2^)], *wR*(*F* ^2^), *S*	0.059, 0.166, 1.08
No. of reflections	6140
No. of parameters	408
H-atom treatment	H atoms treated by a mixture of independent and constrained refinement
Δρ_max_, Δρ_min_ (e Å^−3^)	0.34, −0.36

Computer programs: *CrysAlis PRO* (Rigaku OD, 2021 ▸), *SHELXT* (Sheldrick, 2015*a* ▸), *SHELXL* (Sheldrick, 2015*b* ▸), *ORTEP-3 for Windows* (Farrugia, 2012 ▸) and *PLATON* (Spek, 2020 ▸).

## supplementary crystallographic information

### Crystal data

**Table:**

C_32_H_27_N_5_O_2_·C_3_H_7_NO	*F*(000) = 1240
*M**_r_* = 586.68	*D*_x_ = 1.331 Mg m^−^^3^
Monoclinic, *P*2_1_/*c*	Cu *K*α radiation, λ = 1.54184 Å
*a* = 17.6747 (3) Å	Cell parameters from 19771 reflections
*b* = 15.7656 (2) Å	θ = 3.8–79.0°
*c* = 10.9086 (2) Å	µ = 0.70 mm^−^^1^
β = 105.666 (2)°	*T* = 100 K
*V* = 2926.79 (9) Å^3^	Prism, colourless
*Z* = 4	0.14 × 0.11 × 0.08 mm

### Data collection

**Table:**

XtaLAB Synergy, Dualflex, HyPix diffractometer	5275 reflections with *I* > 2σ(*I*)
Radiation source: micro-focus sealed X-ray tube	*R*_int_ = 0.051
φ and ω scans	θ_max_ = 79.5°, θ_min_ = 3.8°
Absorption correction: multi-scan (CrysAlisPro; Rigaku OD, 2021)	*h* = −22→21
*T*_min_ = 0.900, *T*_max_ = 0.936	*k* = −20→19
31226 measured reflections	*l* = −12→13
6140 independent reflections	

### Refinement

**Table:**

Refinement on *F*^2^	0 restraints
Least-squares matrix: full	Hydrogen site location: mixed
*R*[*F*^2^ > 2σ(*F*^2^)] = 0.059	H atoms treated by a mixture of independent and constrained refinement
*wR*(*F*^2^) = 0.166	*w* = 1/[σ^2^(*F*_o_^2^) + (0.0745*P*)^2^ + 3.3477*P*] where *P* = (*F*_o_^2^ + 2*F*_c_^2^)/3
*S* = 1.08	(Δ/σ)_max_ < 0.001
6140 reflections	Δρ_max_ = 0.34 e Å^−^^3^
408 parameters	Δρ_min_ = −0.36 e Å^−^^3^

### Special details

**Table:**

Geometry. All esds (except the esd in the dihedral angle between two l.s. planes) are estimated using the full covariance matrix. The cell esds are taken into account individually in the estimation of esds in distances, angles and torsion angles; correlations between esds in cell parameters are only used when they are defined by crystal symmetry. An approximate (isotropic) treatment of cell esds is used for estimating esds involving l.s. planes.
Refinement. Owing to poor agreement between observed and calculated intensities, twenty-three outliers (2 5 1, 3 8 1, 1 8 9, 12 2 8, 1 8 1, 4 0 8, 2 1 1, 4 12 10, 4 2 7, 5 12 8, 1 1 7, 3 6 8, 6 3 9, 1 9 9, 12 11 1, 2 9 9, 1 9 7, 5 3 9, 5 14 7, 3 6 1, 0 1 7, 12 2 7, and 1 11 8) were omitted in the final cycles of refinement.

### Fractional atomic coordinates and isotropic or equivalent isotropic displacement parameters (Å^2^)

**Table:**

	*x*	*y*	*z*	*U*_iso_*/*U*_eq_	
C1	0.84688 (12)	0.49523 (12)	0.84606 (19)	0.0192 (4)	
C2	0.90039 (12)	0.46714 (13)	0.76567 (19)	0.0198 (4)	
C4	0.81629 (12)	0.33885 (13)	0.70834 (19)	0.0206 (4)	
C5	0.77485 (12)	0.35958 (13)	0.81000 (19)	0.0194 (4)	
H5	0.766607	0.305061	0.851447	0.023*	
C6	0.69240 (12)	0.39782 (13)	0.74590 (19)	0.0203 (4)	
H6	0.682604	0.393814	0.651413	0.024*	
C7	0.69769 (12)	0.49505 (13)	0.78496 (19)	0.0200 (4)	
C8	0.77028 (12)	0.53623 (13)	0.75358 (19)	0.0195 (4)	
H8	0.769058	0.518255	0.665155	0.023*	
C9	0.82223 (12)	0.41853 (13)	0.91434 (19)	0.0198 (4)	
C10	0.70680 (12)	0.49526 (13)	0.9264 (2)	0.0207 (4)	
C11	0.88965 (12)	0.55627 (13)	0.94255 (19)	0.0201 (4)	
C12	0.91367 (12)	0.37153 (13)	0.5957 (2)	0.0214 (4)	
C13	0.87775 (13)	0.39897 (14)	0.4729 (2)	0.0261 (5)	
H13	0.829930	0.429983	0.455287	0.031*	
C14	0.91246 (15)	0.38060 (16)	0.3761 (2)	0.0305 (5)	
H14	0.888170	0.398723	0.291590	0.037*	
C15	0.98255 (14)	0.33583 (15)	0.4026 (2)	0.0283 (5)	
H15	1.006297	0.323451	0.336214	0.034*	
C16	1.01812 (13)	0.30904 (14)	0.5263 (2)	0.0271 (5)	
H16	1.066067	0.278253	0.544171	0.033*	
C17	0.98380 (13)	0.32713 (13)	0.6236 (2)	0.0241 (4)	
H17	1.008099	0.309285	0.708266	0.029*	
C18	0.62485 (12)	0.35539 (13)	0.7832 (2)	0.0208 (4)	
C19	0.63465 (13)	0.31443 (13)	0.9005 (2)	0.0225 (4)	
H19	0.685990	0.306811	0.955333	0.027*	
C20	0.57009 (13)	0.28484 (14)	0.9374 (2)	0.0241 (4)	
H20	0.578280	0.256282	1.016577	0.029*	
C21	0.49370 (13)	0.29599 (14)	0.8612 (2)	0.0248 (5)	
C22	0.48456 (13)	0.33204 (14)	0.7412 (2)	0.0249 (5)	
H22	0.433380	0.336973	0.684802	0.030*	
C23	0.54892 (13)	0.36093 (13)	0.7026 (2)	0.0231 (4)	
H23	0.541005	0.384761	0.620081	0.028*	
C24	0.42432 (13)	0.27319 (15)	0.9096 (2)	0.0288 (5)	
H24A	0.408264	0.322797	0.950610	0.043*	
H24B	0.438846	0.226951	0.971608	0.043*	
H24C	0.380636	0.254957	0.838265	0.043*	
C25	0.62564 (13)	0.53826 (13)	0.7117 (2)	0.0231 (4)	
C26	0.77331 (12)	0.63271 (13)	0.75573 (19)	0.0213 (4)	
C27	0.74616 (13)	0.68268 (13)	0.8408 (2)	0.0229 (4)	
H27	0.721083	0.656657	0.897947	0.028*	
C28	0.75561 (13)	0.77005 (14)	0.8423 (2)	0.0244 (4)	
H28	0.736015	0.802987	0.899962	0.029*	
C29	0.79307 (13)	0.81081 (14)	0.7616 (2)	0.0242 (4)	
C30	0.81945 (13)	0.76077 (14)	0.6766 (2)	0.0251 (5)	
H30	0.844772	0.786878	0.619812	0.030*	
C31	0.80946 (13)	0.67318 (14)	0.6730 (2)	0.0238 (4)	
H31	0.827533	0.640525	0.613376	0.029*	
C32	0.80575 (15)	0.90535 (14)	0.7689 (2)	0.0301 (5)	
H32A	0.852432	0.918575	0.838280	0.045*	
H32B	0.759739	0.933163	0.784828	0.045*	
H32C	0.813314	0.925827	0.688169	0.045*	
C33	0.89004 (12)	0.37404 (13)	1.0063 (2)	0.0218 (4)	
H33A	0.919202	0.414849	1.069304	0.033*	
H33B	0.925089	0.349923	0.959485	0.033*	
H33C	0.869776	0.328506	1.049816	0.033*	
C34	0.63579 (14)	0.47173 (15)	0.3770 (2)	0.0275 (5)	
H34	0.593630	0.505419	0.388302	0.033*	
C35	0.68357 (15)	0.37063 (15)	0.2489 (2)	0.0300 (5)	
H35A	0.709455	0.394672	0.187861	0.045*	
H35B	0.658547	0.316752	0.215915	0.045*	
H35C	0.722664	0.360780	0.330382	0.045*	
C36	0.54932 (14)	0.43254 (18)	0.1702 (2)	0.0338 (5)	
H36A	0.521478	0.378714	0.169526	0.051*	
H36B	0.558662	0.441861	0.086657	0.051*	
H36C	0.517414	0.479056	0.188991	0.051*	
N2	0.95780 (11)	0.51308 (12)	0.75995 (18)	0.0242 (4)	
H2N	0.9840 (16)	0.4923 (18)	0.702 (3)	0.029*	
N3	0.87658 (10)	0.39156 (11)	0.69547 (16)	0.0205 (4)	
N10	0.66000 (11)	0.52902 (12)	0.98087 (19)	0.0252 (4)	
H10N	0.6751 (16)	0.5206 (18)	1.071 (3)	0.030*	
N11	0.92084 (11)	0.60155 (12)	1.02287 (17)	0.0250 (4)	
N25	0.57038 (11)	0.56806 (13)	0.64654 (19)	0.0298 (4)	
N34	0.62423 (11)	0.42955 (12)	0.26736 (18)	0.0265 (4)	
O4	0.79621 (9)	0.27759 (10)	0.63952 (15)	0.0270 (4)	
O9	0.77226 (8)	0.45054 (9)	0.99082 (13)	0.0202 (3)	
O34	0.69653 (10)	0.47051 (11)	0.46440 (15)	0.0332 (4)	

### Atomic displacement parameters (Å^2^)

**Table:**

	*U*^11^	*U*^22^	*U*^33^	*U*^12^	*U*^13^	*U*^23^
C1	0.0237 (10)	0.0151 (9)	0.0192 (9)	−0.0017 (7)	0.0064 (8)	−0.0003 (7)
C2	0.0234 (10)	0.0172 (9)	0.0190 (9)	0.0005 (8)	0.0063 (8)	0.0001 (8)
C4	0.0235 (10)	0.0171 (9)	0.0218 (10)	0.0008 (8)	0.0069 (8)	0.0007 (8)
C5	0.0240 (10)	0.0146 (9)	0.0209 (10)	−0.0016 (7)	0.0080 (8)	−0.0012 (7)
C6	0.0245 (10)	0.0162 (9)	0.0198 (10)	0.0002 (8)	0.0052 (8)	−0.0017 (7)
C7	0.0219 (10)	0.0168 (9)	0.0210 (10)	0.0003 (8)	0.0052 (8)	0.0000 (8)
C8	0.0248 (10)	0.0178 (9)	0.0166 (9)	−0.0004 (8)	0.0064 (8)	−0.0003 (7)
C9	0.0231 (10)	0.0172 (9)	0.0207 (10)	0.0000 (8)	0.0086 (8)	−0.0010 (8)
C10	0.0239 (10)	0.0148 (9)	0.0235 (10)	−0.0018 (7)	0.0066 (8)	−0.0002 (8)
C11	0.0242 (10)	0.0164 (9)	0.0209 (10)	0.0007 (8)	0.0080 (8)	0.0000 (8)
C12	0.0273 (10)	0.0176 (9)	0.0210 (10)	−0.0034 (8)	0.0092 (8)	−0.0031 (8)
C13	0.0284 (11)	0.0244 (11)	0.0260 (11)	0.0021 (9)	0.0081 (9)	0.0000 (9)
C14	0.0384 (13)	0.0314 (12)	0.0233 (11)	−0.0003 (10)	0.0110 (9)	0.0015 (9)
C15	0.0363 (12)	0.0256 (11)	0.0282 (11)	−0.0051 (9)	0.0179 (9)	−0.0069 (9)
C16	0.0278 (11)	0.0213 (10)	0.0346 (12)	−0.0010 (9)	0.0126 (9)	−0.0039 (9)
C17	0.0282 (11)	0.0184 (10)	0.0262 (11)	−0.0007 (8)	0.0082 (8)	−0.0009 (8)
C18	0.0242 (10)	0.0151 (9)	0.0237 (10)	−0.0001 (8)	0.0078 (8)	−0.0037 (8)
C19	0.0243 (10)	0.0196 (10)	0.0224 (10)	−0.0005 (8)	0.0046 (8)	−0.0012 (8)
C20	0.0303 (11)	0.0212 (10)	0.0216 (10)	−0.0029 (8)	0.0083 (9)	−0.0013 (8)
C21	0.0282 (11)	0.0191 (10)	0.0285 (11)	−0.0030 (8)	0.0100 (9)	−0.0049 (8)
C22	0.0238 (10)	0.0216 (10)	0.0274 (11)	−0.0006 (8)	0.0035 (8)	−0.0026 (8)
C23	0.0298 (11)	0.0178 (10)	0.0213 (10)	−0.0009 (8)	0.0062 (8)	−0.0003 (8)
C24	0.0274 (11)	0.0276 (12)	0.0328 (12)	−0.0032 (9)	0.0103 (9)	−0.0026 (9)
C25	0.0265 (11)	0.0188 (10)	0.0254 (11)	−0.0018 (8)	0.0093 (9)	−0.0021 (8)
C26	0.0241 (10)	0.0181 (10)	0.0203 (10)	0.0002 (8)	0.0037 (8)	0.0018 (8)
C27	0.0287 (11)	0.0184 (10)	0.0228 (10)	0.0001 (8)	0.0088 (8)	0.0012 (8)
C28	0.0307 (11)	0.0194 (10)	0.0223 (10)	0.0020 (8)	0.0059 (9)	−0.0016 (8)
C29	0.0282 (11)	0.0189 (10)	0.0224 (10)	−0.0004 (8)	0.0018 (8)	0.0019 (8)
C30	0.0324 (11)	0.0199 (10)	0.0230 (10)	−0.0028 (9)	0.0073 (9)	0.0034 (8)
C31	0.0300 (11)	0.0210 (10)	0.0213 (10)	0.0004 (8)	0.0084 (8)	0.0003 (8)
C32	0.0392 (13)	0.0185 (10)	0.0317 (12)	−0.0009 (9)	0.0076 (10)	0.0021 (9)
C33	0.0259 (10)	0.0173 (9)	0.0220 (10)	−0.0006 (8)	0.0060 (8)	0.0014 (8)
C34	0.0314 (11)	0.0279 (11)	0.0243 (11)	0.0010 (9)	0.0094 (9)	−0.0010 (9)
C35	0.0376 (13)	0.0254 (11)	0.0277 (11)	0.0027 (9)	0.0100 (10)	−0.0009 (9)
C36	0.0295 (12)	0.0434 (14)	0.0277 (12)	0.0001 (10)	0.0063 (9)	−0.0046 (10)
N2	0.0280 (9)	0.0221 (9)	0.0245 (9)	−0.0027 (7)	0.0105 (8)	−0.0021 (7)
N3	0.0255 (9)	0.0163 (8)	0.0217 (8)	−0.0020 (7)	0.0098 (7)	−0.0027 (7)
N10	0.0311 (10)	0.0216 (9)	0.0260 (10)	−0.0001 (7)	0.0131 (8)	0.0001 (7)
N11	0.0283 (9)	0.0212 (9)	0.0255 (9)	−0.0004 (7)	0.0073 (7)	−0.0010 (7)
N25	0.0291 (10)	0.0244 (10)	0.0346 (11)	0.0011 (8)	0.0061 (8)	0.0030 (8)
N34	0.0288 (10)	0.0263 (10)	0.0241 (9)	0.0010 (8)	0.0064 (8)	−0.0018 (7)
O4	0.0322 (8)	0.0205 (7)	0.0307 (8)	−0.0034 (6)	0.0126 (7)	−0.0073 (6)
O9	0.0241 (7)	0.0187 (7)	0.0191 (7)	0.0014 (5)	0.0081 (6)	0.0003 (5)
O34	0.0358 (9)	0.0365 (9)	0.0253 (8)	0.0032 (7)	0.0049 (7)	−0.0042 (7)

### Geometric parameters (Å, º)

**Table:**

C1—C11	1.475 (3)	C20—C21	1.392 (3)
C1—C2	1.519 (3)	C20—H20	0.9500
C1—C9	1.543 (3)	C21—C22	1.396 (3)
C1—C8	1.591 (3)	C21—C24	1.504 (3)
C2—N2	1.262 (3)	C22—C23	1.392 (3)
C2—N3	1.418 (3)	C22—H22	0.9500
C4—O4	1.216 (3)	C23—H23	0.9500
C4—N3	1.388 (3)	C24—H24A	0.9800
C4—C5	1.520 (3)	C24—H24B	0.9800
C5—C9	1.532 (3)	C24—H24C	0.9800
C5—C6	1.557 (3)	C25—N25	1.143 (3)
C5—H5	1.0000	C26—C31	1.393 (3)
C6—C18	1.517 (3)	C26—C27	1.397 (3)
C6—C7	1.587 (3)	C27—C28	1.387 (3)
C6—H6	1.0000	C27—H27	0.9500
C7—C25	1.475 (3)	C28—C29	1.393 (3)
C7—C10	1.507 (3)	C28—H28	0.9500
C7—C8	1.556 (3)	C29—C30	1.391 (3)
C8—C26	1.522 (3)	C29—C32	1.506 (3)
C8—H8	1.0000	C30—C31	1.391 (3)
C9—O9	1.459 (2)	C30—H30	0.9500
C9—C33	1.512 (3)	C31—H31	0.9500
C10—N10	1.259 (3)	C32—H32A	0.9800
C10—O9	1.375 (2)	C32—H32B	0.9800
C11—N11	1.149 (3)	C32—H32C	0.9800
C12—C17	1.384 (3)	C33—H33A	0.9800
C12—C13	1.388 (3)	C33—H33B	0.9800
C12—N3	1.449 (3)	C33—H33C	0.9800
C13—C14	1.387 (3)	C34—O34	1.229 (3)
C13—H13	0.9500	C34—N34	1.335 (3)
C14—C15	1.387 (3)	C34—H34	0.9500
C14—H14	0.9500	C35—N34	1.455 (3)
C15—C16	1.392 (3)	C35—H35A	0.9800
C15—H15	0.9500	C35—H35B	0.9800
C16—C17	1.387 (3)	C35—H35C	0.9800
C16—H16	0.9500	C36—N34	1.456 (3)
C17—H17	0.9500	C36—H36A	0.9800
C18—C23	1.395 (3)	C36—H36B	0.9800
C18—C19	1.401 (3)	C36—H36C	0.9800
C19—C20	1.389 (3)	N2—H2N	0.94 (3)
C19—H19	0.9500	N10—H10N	0.95 (3)
			
C11—C1—C2	108.85 (17)	C21—C20—H20	119.2
C11—C1—C9	108.90 (17)	C20—C21—C22	117.3 (2)
C2—C1—C9	110.55 (16)	C20—C21—C24	120.8 (2)
C11—C1—C8	111.83 (16)	C22—C21—C24	121.8 (2)
C2—C1—C8	107.95 (16)	C23—C22—C21	121.3 (2)
C9—C1—C8	108.76 (16)	C23—C22—H22	119.3
N2—C2—N3	125.53 (19)	C21—C22—H22	119.3
N2—C2—C1	119.71 (19)	C22—C23—C18	120.9 (2)
N3—C2—C1	114.63 (17)	C22—C23—H23	119.5
O4—C4—N3	121.14 (19)	C18—C23—H23	119.5
O4—C4—C5	120.22 (18)	C21—C24—H24A	109.5
N3—C4—C5	118.64 (17)	C21—C24—H24B	109.5
C4—C5—C9	113.46 (17)	H24A—C24—H24B	109.5
C4—C5—C6	109.41 (16)	C21—C24—H24C	109.5
C9—C5—C6	110.87 (16)	H24A—C24—H24C	109.5
C4—C5—H5	107.6	H24B—C24—H24C	109.5
C9—C5—H5	107.6	N25—C25—C7	174.4 (2)
C6—C5—H5	107.6	C31—C26—C27	118.15 (19)
C18—C6—C5	114.69 (17)	C31—C26—C8	117.91 (19)
C18—C6—C7	110.33 (16)	C27—C26—C8	123.83 (19)
C5—C6—C7	105.86 (16)	C28—C27—C26	120.4 (2)
C18—C6—H6	108.6	C28—C27—H27	119.8
C5—C6—H6	108.6	C26—C27—H27	119.8
C7—C6—H6	108.6	C27—C28—C29	121.8 (2)
C25—C7—C10	113.04 (18)	C27—C28—H28	119.1
C25—C7—C8	109.31 (17)	C29—C28—H28	119.1
C10—C7—C8	110.95 (17)	C30—C29—C28	117.5 (2)
C25—C7—C6	108.74 (16)	C30—C29—C32	121.6 (2)
C10—C7—C6	105.03 (16)	C28—C29—C32	120.8 (2)
C8—C7—C6	109.64 (16)	C29—C30—C31	121.2 (2)
C26—C8—C7	116.18 (17)	C29—C30—H30	119.4
C26—C8—C1	112.08 (16)	C31—C30—H30	119.4
C7—C8—C1	107.58 (16)	C30—C31—C26	120.9 (2)
C26—C8—H8	106.8	C30—C31—H31	119.6
C7—C8—H8	106.8	C26—C31—H31	119.6
C1—C8—H8	106.8	C29—C32—H32A	109.5
O9—C9—C33	105.95 (16)	C29—C32—H32B	109.5
O9—C9—C5	109.93 (16)	H32A—C32—H32B	109.5
C33—C9—C5	112.84 (17)	C29—C32—H32C	109.5
O9—C9—C1	107.35 (16)	H32A—C32—H32C	109.5
C33—C9—C1	114.04 (17)	H32B—C32—H32C	109.5
C5—C9—C1	106.61 (16)	C9—C33—H33A	109.5
N10—C10—O9	123.02 (19)	C9—C33—H33B	109.5
N10—C10—C7	125.6 (2)	H33A—C33—H33B	109.5
O9—C10—C7	111.38 (17)	C9—C33—H33C	109.5
N11—C11—C1	175.8 (2)	H33A—C33—H33C	109.5
C17—C12—C13	121.3 (2)	H33B—C33—H33C	109.5
C17—C12—N3	120.32 (19)	O34—C34—N34	125.3 (2)
C13—C12—N3	118.38 (19)	O34—C34—H34	117.3
C14—C13—C12	119.2 (2)	N34—C34—H34	117.3
C14—C13—H13	120.4	N34—C35—H35A	109.5
C12—C13—H13	120.4	N34—C35—H35B	109.5
C15—C14—C13	120.1 (2)	H35A—C35—H35B	109.5
C15—C14—H14	119.9	N34—C35—H35C	109.5
C13—C14—H14	119.9	H35A—C35—H35C	109.5
C14—C15—C16	120.0 (2)	H35B—C35—H35C	109.5
C14—C15—H15	120.0	N34—C36—H36A	109.5
C16—C15—H15	120.0	N34—C36—H36B	109.5
C17—C16—C15	120.2 (2)	H36A—C36—H36B	109.5
C17—C16—H16	119.9	N34—C36—H36C	109.5
C15—C16—H16	119.9	H36A—C36—H36C	109.5
C12—C17—C16	119.1 (2)	H36B—C36—H36C	109.5
C12—C17—H17	120.4	C2—N2—H2N	112.5 (17)
C16—C17—H17	120.4	C4—N3—C2	124.93 (17)
C23—C18—C19	117.73 (19)	C4—N3—C12	117.45 (17)
C23—C18—C6	119.75 (19)	C2—N3—C12	117.42 (17)
C19—C18—C6	122.41 (18)	C10—N10—H10N	112.8 (17)
C20—C19—C18	120.7 (2)	C34—N34—C35	120.03 (19)
C20—C19—H19	119.7	C34—N34—C36	121.7 (2)
C18—C19—H19	119.7	C35—N34—C36	117.86 (19)
C19—C20—C21	121.7 (2)	C10—O9—C9	116.16 (15)
C19—C20—H20	119.2		
			
C11—C1—C2—N2	−21.8 (3)	N3—C12—C13—C14	179.8 (2)
C9—C1—C2—N2	−141.3 (2)	C12—C13—C14—C15	−0.5 (3)
C8—C1—C2—N2	99.8 (2)	C13—C14—C15—C16	0.2 (4)
C11—C1—C2—N3	162.27 (17)	C14—C15—C16—C17	−0.2 (3)
C9—C1—C2—N3	42.7 (2)	C13—C12—C17—C16	−0.7 (3)
C8—C1—C2—N3	−76.1 (2)	N3—C12—C17—C16	−179.82 (19)
O4—C4—C5—C9	161.19 (19)	C15—C16—C17—C12	0.4 (3)
N3—C4—C5—C9	−19.4 (3)	C5—C6—C18—C23	−156.70 (18)
O4—C4—C5—C6	−74.4 (2)	C7—C6—C18—C23	83.9 (2)
N3—C4—C5—C6	105.0 (2)	C5—C6—C18—C19	27.2 (3)
C4—C5—C6—C18	128.31 (18)	C7—C6—C18—C19	−92.2 (2)
C9—C5—C6—C18	−105.81 (19)	C23—C18—C19—C20	−3.6 (3)
C4—C5—C6—C7	−109.82 (18)	C6—C18—C19—C20	172.57 (19)
C9—C5—C6—C7	16.1 (2)	C18—C19—C20—C21	−1.2 (3)
C18—C6—C7—C25	−64.0 (2)	C19—C20—C21—C22	5.1 (3)
C5—C6—C7—C25	171.40 (16)	C19—C20—C21—C24	−172.4 (2)
C18—C6—C7—C10	57.3 (2)	C20—C21—C22—C23	−4.2 (3)
C5—C6—C7—C10	−67.34 (19)	C24—C21—C22—C23	173.3 (2)
C18—C6—C7—C8	176.56 (16)	C21—C22—C23—C18	−0.5 (3)
C5—C6—C7—C8	51.9 (2)	C19—C18—C23—C22	4.4 (3)
C25—C7—C8—C26	47.0 (2)	C6—C18—C23—C22	−171.82 (19)
C10—C7—C8—C26	−78.3 (2)	C7—C8—C26—C31	−148.55 (19)
C6—C7—C8—C26	166.10 (16)	C1—C8—C26—C31	87.2 (2)
C25—C7—C8—C1	173.51 (16)	C7—C8—C26—C27	35.4 (3)
C10—C7—C8—C1	48.2 (2)	C1—C8—C26—C27	−88.9 (2)
C6—C7—C8—C1	−67.4 (2)	C31—C26—C27—C28	−0.3 (3)
C11—C1—C8—C26	19.3 (2)	C8—C26—C27—C28	175.78 (19)
C2—C1—C8—C26	−100.43 (19)	C26—C27—C28—C29	−1.0 (3)
C9—C1—C8—C26	139.59 (17)	C27—C28—C29—C30	1.4 (3)
C11—C1—C8—C7	−109.61 (19)	C27—C28—C29—C32	−177.4 (2)
C2—C1—C8—C7	130.67 (17)	C28—C29—C30—C31	−0.6 (3)
C9—C1—C8—C7	10.7 (2)	C32—C29—C30—C31	178.2 (2)
C4—C5—C9—O9	167.19 (16)	C29—C30—C31—C26	−0.7 (3)
C6—C5—C9—O9	43.6 (2)	C27—C26—C31—C30	1.1 (3)
C4—C5—C9—C33	−74.8 (2)	C8—C26—C31—C30	−175.21 (19)
C6—C5—C9—C33	161.62 (17)	O4—C4—N3—C2	175.69 (19)
C4—C5—C9—C1	51.1 (2)	C5—C4—N3—C2	−3.7 (3)
C6—C5—C9—C1	−72.5 (2)	O4—C4—N3—C12	1.0 (3)
C11—C1—C9—O9	59.6 (2)	C5—C4—N3—C12	−178.45 (17)
C2—C1—C9—O9	179.10 (15)	N2—C2—N3—C4	175.9 (2)
C8—C1—C9—O9	−62.54 (19)	C1—C2—N3—C4	−8.5 (3)
C11—C1—C9—C33	−57.5 (2)	N2—C2—N3—C12	−9.4 (3)
C2—C1—C9—C33	62.1 (2)	C1—C2—N3—C12	166.25 (17)
C8—C1—C9—C33	−179.57 (16)	C17—C12—N3—C4	−98.1 (2)
C11—C1—C9—C5	177.32 (16)	C13—C12—N3—C4	82.8 (2)
C2—C1—C9—C5	−63.1 (2)	C17—C12—N3—C2	86.7 (2)
C8—C1—C9—C5	55.2 (2)	C13—C12—N3—C2	−92.4 (2)
C25—C7—C10—N10	−1.0 (3)	O34—C34—N34—C35	5.8 (4)
C8—C7—C10—N10	122.2 (2)	O34—C34—N34—C36	178.1 (2)
C6—C7—C10—N10	−119.4 (2)	N10—C10—O9—C9	−177.47 (19)
C25—C7—C10—O9	176.72 (16)	C7—C10—O9—C9	4.7 (2)
C8—C7—C10—O9	−60.1 (2)	C33—C9—O9—C10	178.80 (16)
C6—C7—C10—O9	58.3 (2)	C5—C9—O9—C10	−59.0 (2)
C17—C12—C13—C14	0.8 (3)	C1—C9—O9—C10	56.6 (2)

### Hydrogen-bond geometry (Å, º)

*Cg*7 is the centroid of the C26–C31 benzene ring.

**Table:**

*D*—H···*A*	*D*—H	H···*A*	*D*···*A*	*D*—H···*A*
C6—H6···O34	1.00	2.44	3.297 (3)	143
C8—H8···O34	1.00	2.34	3.241 (3)	149
C13—H13···O34	0.95	2.47	3.373 (3)	159
C27—H27···N10	0.95	2.56	3.432 (3)	152
C30—H30···N11^i^	0.95	2.61	3.515 (3)	160
C33—H33*C*···O4^ii^	0.98	2.48	3.447 (3)	170
C35—H35*C*···O34	0.98	2.39	2.787 (3)	104
C24—H24*C*···*Cg*7^iii^	0.98	2.84	3.715 (2)	148

Symmetry codes: (i) *x*, −*y*+3/2, *z*−1/2; (ii) *x*, −*y*+1/2, *z*+1/2; (iii) −*x*+1, *y*−1/2, −*z*+3/2.

## References

[bb1] Aliyeva, K. N., Maharramov, A. M., Allahverdiyev, M. A., Gurbanov, A. V. & Brito, I. (2011). *Acta Cryst.* E**67**, o2293. 10.1107/S160053681103145X PMC3200700 22058933

[bb2] Bernstein, J., Davis, R. E., Shimoni, L. & Chang, N.-L. (1995). *Angew. Chem. Int. Ed. Engl.* **34**, 1555–1573.

[bb3] Cremer, D. & Pople, J. A. (1975). *J. Am. Chem. Soc.* **97**, 1354–1358.

[bb4] Farrugia, L. J. (2012). *J. Appl. Cryst.* **45**, 849–854.

[bb5] Girgis, A. S., Saleh, D. O., George, R. F., Srour, A. M., Pillai, G. G., Panda, C. S. & Katritzky, A. R. (2015). *Eur. J. Med. Chem.* **89**, 835–843. 10.1016/j.ejmech.2013.12.032 25462283

[bb6] Groom, C. R., Bruno, I. J., Lightfoot, M. P. & Ward, S. C. (2016). *Acta Cryst.* B**72**, 171–179. 10.1107/S2052520616003954 PMC4822653 27048719

[bb7] Hathwar, V. R., Sist, M., Jørgensen, M. R. V., Mamakhel, A. H., Wang, X., Hoffmann, C. M., Sugimoto, K., Overgaard, J. & Iversen, B. B. (2015). *IUCrJ*, **2**, 563–574. 10.1107/S2052252515012130 PMC4547824 26306198

[bb8] Hulme, A. T., Fernandes, P., Florence, A., Johnston, A. & Shankland, K. (2006). *Acta Cryst.* E**62**, o3046–o3048.

[bb9] Khalilov, A. N., Khrustalev, V. N., Tereshina, T. A., Akkurt, M., Rzayev, R. M., Akobirshoeva, A. A. & Mamedov, İ. G. (2022). *Acta Cryst.* E**78**, 525–529. 10.1107/S2056989022004297 PMC9069515 35547793

[bb10] Kumaran, D., Ponnuswamy, M. N., Shanmugam, G., Ponnuswamy, S., Jeyaraman, R., Shivakumar, K. & Fun, H. K. (1999). *Acta Cryst.* B**55**, 793–798. 10.1107/s0108768199005273 10927419

[bb11] Kumari, P., Narayana, C., Dubey, S., Gupta, A. & Sagar, R. (2018). *Org. Biomol. Chem.* **16**, 2049–2059. 10.1039/c7ob03186f 29411817

[bb12] Mamedov, I. G., Khrustalev, V. N., Akkurt, M., Novikov, A. P., Asgarova, A. R., Aliyeva, K. N. & Akobirshoeva, A. A. (2022). *Acta Cryst.* E**78**, 291–296. 10.1107/S2056989022001232 PMC8900508 35371550

[bb13] Mamedov, I. G., Khrustalev, V. N., Dorovatovskii, P. V., Naghiev, F. N. & Maharramov, A. M. (2019). *Mendeleev Commun.* **29**, 232–233.

[bb14] Naghiyev, F. N., Akkurt, M., Askerov, R. K., Mamedov, I. G., Rzayev, R. M., Chyrka, T. & Maharramov, A. M. (2020). *Acta Cryst.* E**76**, 720–723. 10.1107/S2056989020005381 PMC7199244 32431939

[bb15] Naghiyev, F. N., Khrustalev, V. N., Novikov, A. P., Akkurt, M., Rzayev, R. M., Akobirshoeva, A. A. & Mamedov, I. G. (2022). *Acta Cryst.* E**78**, 554–558. 10.1107/S2056989022004741 PMC9431780 36072149

[bb16] Naghiyev, F. N., Tereshina, T. A., Khrustalev, V. N., Akkurt, M., Rzayev, R. M., Akobirshoeva, A. A. & Mamedov, İ. G. (2021). *Acta Cryst.* E**77**, 516–521. 10.1107/S2056989021003583 PMC8100256 34026256

[bb17] Rigaku OD (2021). *CrysAlis PRO*. Rigaku Oxford Diffraction, Yarnton, England.

[bb18] Sheldrick, G. M. (2015*a*). *Acta Cryst.* A**71**, 3–8.

[bb19] Sheldrick, G. M. (2015*b*). *Acta Cryst.* C**71**, 3–8.

[bb20] Spek, A. L. (2020). *Acta Cryst.* E**76**, 1–11. 10.1107/S2056989019016244 PMC6944088 31921444

[bb21] Turner, M. J., McKinnon, J. J., Wolff, S. K., Grimwood, D. J., Spackman, P. R., Jayatilaka, D. & Spackman, M. A. (2017). *CrystalExplorer17*. The University of Western Australia.

[bb22] Venkateswaramoorthi, R., Rizwana Begum, S., Hema, R., Krishnasamy, K. & Anitha, A. G. (2013). *Acta Cryst.* E**69**, o768. 10.1107/S1600536813010180 PMC3648293 23723913

[bb23] Viswanathan, A., Kute, D., Musa, A., Konda Mani, S., Sipilä, V., Emmert-Streib, F., Zubkov, F. I., Gurbanov, A. V., Yli-Harja, O. & Kandhavelu, M. (2019). *Eur. J. Med. Chem.* **166**, 291–303. 10.1016/j.ejmech.2019.01.021 30731398

[bb24] Zubkov, F. I., Mertsalov, D. F., Zaytsev, V. P., Varlamov, A. V., Gurbanov, A. V., Dorovatovskii, P. V., Timofeeva, T. V., Khrustalev, V. N. & Mahmudov, K. T. (2018). *J. Mol. Liq.* **249**, 949–952.

